# Escape room-based learning for HPV vaccine education in Japanese female university students: a controlled, three-group study

**DOI:** 10.1038/s41598-026-48126-w

**Published:** 2026-04-18

**Authors:** Keita Kondo, Kosuke Ishizuka, Taiju Miyagami, Mitsuki Ugajin, Miyuki Yuasa, Mizue Saita, Toshio Naito

**Affiliations:** 1 Department of General Medicine, Faculty of Medicine, Juntendo University, Tokyo, Japan; 2 Department of General Medicine, Yokohama City University School of Medicine, Yokohama, Japan; 3 Shiga University of Medical Science School, Otsu, Japan; 4 Kyoritsu Women’s University Student Support Office Health Room, Tokyo, Japan

**Keywords:** HPV vaccination, Escape room, Vaccine education, Game-based learning, Vaccination intention, Health care, Psychology, Psychology

## Abstract

Japan’s human papillomavirus (HPV) vaccine coverage remains low, highlighting the need for engaging education for young adults. An escape-room intervention, a traditional lecture, and a non-intervention control were compared in Japanese female university students. Questionnaires were completed at baseline, immediately post-intervention, and three months later. Knowledge and vaccination intention were assessed at all time points; satisfaction and perceived comprehension were assessed immediately post-intervention; and HPV vaccination initiation was assessed at three months. Open-ended responses from the escape-room group were analyzed using quantitative content analysis based on Fink’s taxonomy. For the 267 students (escape room *n* = 92; lecture *n* = 75; control *n* = 100), the lecture produced greater immediate gains in knowledge and intention. At three months, both intervention groups retained higher knowledge than the control group, with no difference between escape room and lecture. In participants unvaccinated at baseline (0 doses), vaccination intention increased more from baseline to three months in the escape-room group than in the control group, whereas vaccination initiation was significantly higher in the lecture group. Qualitative findings suggested the escape room promoted emotional engagement, intrinsic motivation, and peer collaboration. Escape rooms may complement traditional vaccine education by providing an interactive, low-resource format that supports learning and motivation.

**Supplementary Information:**

The online version contains supplementary material available at 10.1038/s41598-026-48126-w.

## Introduction

The human papillomavirus (HPV) vaccine is highly effective in preventing cervical cancer. The Advisory Committee on Immunization Practices (ACIP) recommends starting vaccination at ages 11–12 years and completing it before age 15 years^1^. Although HPV vaccination has been introduced globally, vaccination rates vary considerably by region^2^, underscoring the importance of educational interventions. In Japan, routine HPV vaccination subsidized by public funds was started in 2010 for girls aged 13 to 16 years^3^. However, following reports of various adverse events in recipients, the Ministry of Health, Labour and Welfare suspended its active recommendation in June 2013^3^. Consequently, HPV vaccination coverage in Japan decreased sharply^4^. In 2018, only 0.3% of 15-year-old girls had completed the three-dose schedule, an exceptionally low rate even among developed countries^2^. Subsequently, based on accumulating scientific evidence and expert evaluation, active recommendation for HPV vaccination was reinstated in April 2022^3^. A national catch-up program was launched to provide free vaccination until March 2025 for women born between April 2, 1997, and April 1, 2008, who had been eligible for vaccination during the suspension period^5^. As a result, opportunities for free HPV vaccination were once again expanded. Nevertheless, knowledge and understanding of the HPV vaccine among Japanese youth remain limited. A survey of female university students conducted between 2020 and 2021 showed that the mean correct response rate on a 20-item knowledge test was only 39.6%, and merely 6% of participants expressed an intention to receive the vaccine^6^. Thus, effective vaccine education for young people remains an urgent public health priority in Japan.

In recent years, game-based learning has attracted increasing attention as an educational strategy for promoting health in younger populations. Ou et al. reported in a systematic review that, although game-based approaches to vaccine education are effective in enhancing knowledge, their influence on actual vaccination behavior appears limited^7^. Importantly, most studies included in that review used digital games, whereas evidence for non-digital formats (e.g., paper-based or experiential games) remains extremely scarce.

To address these gaps, a form of experiential learning known as the escape room has recently emerged as a promising educational approach. An escape room is an activity in which participants collaborate to solve puzzles within a limited timeframe to achieve a specific goal^8^. First introduced in Kyoto, Japan, in 2007, escape rooms have since increased in popularity, particularly among younger populations^9^. More recently, the escape room approach has expanded beyond entertainment and has been increasingly used as an educational tool in diverse domains, including medical education and corporate training^10–12^. Through collaborative problem-solving, escape rooms provide a form of experiential learning in which participants can naturally acquire knowledge and enhance their motivation to learn.

Escape rooms have also been recognized as an approach that fosters learner autonomy, teamwork, and communication skills. However, most evaluations of their educational impact have been limited to the “Reaction” level (e.g., satisfaction), with relatively few assessments addressing the “Learning” or “Behavior” levels, such as knowledge acquisition or behavioral change^13^. To the best of our knowledge, no studies to date, either in Japan or internationally, have used escape rooms for HPV vaccine education.

Therefore, the aim of this study was to develop and implement an escape room-based educational program on the HPV vaccine and to evaluate its educational effects both quantitatively and qualitatively, in comparison with a traditional lecture and a non-intervention control group. Specifically, its impacts on knowledge, vaccination intention, and actual vaccination behavior were examined, with the goal of elucidating the potential advantages of the escape room approach in HPV vaccine education.

## Methods

### Ethics statement

This study was conducted in accordance with the Declaration of Helsinki and was approved by the Ethics Review Committee of Juntendo University (approval number: E24-0167; *Evaluation of the effectiveness of HPV (Human Papillomavirus) vaccine education using an escape room*). Prior to participation, students received an oral explanation of the study procedures, after which informed consent was obtained from those who voluntarily agreed with sufficient understanding of the study. It was explicitly stated that participation in this study would have no impact on their academic evaluation. This study was registered in the University Hospital Medical Information Network Clinical Trials Registry (UMIN-CTR; UMIN000056879, first registered on 01/02/2025).

### Study design

To clarify the effects and potential advantages of escape rooms in vaccine education on university students’ knowledge and vaccination behavior, a non-randomized, quasi-experimental study with three conditions and three measurement points, supplemented by quantitative content analysis of open-ended questionnaire responses, was performed.

The quantitative component was a prospective, interventional trial with a three-group comparison, involving students from Kyoritsu Women’s University. Participants were non-randomly allocated to the escape room, lecture, or non-intervention control conditions. Questionnaires were administered before and after the educational interventions to assess class satisfaction, perceived comprehension, intention to receive the HPV vaccine, and scores on a knowledge test. A follow-up survey was conducted three months later to evaluate knowledge retention, vaccination intention, and actual vaccination behavior.

The qualitative component was designed to capture higher-order cognitive and affective outcomes, such as emotional engagement and learner autonomy, that could not be fully assessed through quantitative measures. Open-ended responses were collected and analyzed via content analysis to explore the perceived advantages of the escape room approach for learning about the HPV vaccine.

### Participants

This study was conducted at a single site, Kyoritsu Women’s University, in Japan. The target population comprised biologically female students, with a maximum anticipated enrollment of 300. No restrictions were placed on academic department or age. Participants were recruited via on-campus announcements, and group allocation was not randomized, but was determined at the time of enrollment. To prevent duplicate participation, student identification numbers were checked at registration to ensure that no individual participated in more than one session. Only students who voluntarily consented to participate were included in the study. Once a student had participated, any subsequent participation was not permitted, and duplicate entries were excluded. The withdrawal criterion was defined as any participant’s decision to refuse or revoke consent at any point during the study.

### Procedure

This study was conducted on September 24 and September 26, 2024. Prior to the interventions, posters were displayed on campus stating only that “a class on the HPV vaccine will be held,” without disclosing the specific content or instructional methods. After obtaining ethics committee approval, the schedule was arranged in coordination with the university. Specifically, Group A (escape room group) received its session during the lunch break on September 24. Group B (lecture group) took part in a session after a regular class on September 26. Group C (control group) met during the lunch break on September 26. Students enrolled in one of the scheduled sessions based on their date/time availability and provided in-person informed consent during the pre-class briefing. Therefore, allocation to the escape room, lecture, or control condition was determined by session attendance rather than random assignment. The interventions were implemented as follows.

#### Group A (escape room group)

Participants engaged in a 30-minute escape room activity in teams of three to four, using paper-based materials and a chatbot delivered via LINE (a widely used messaging application in Japan). Although escape rooms are generally designed for teams of four to six participants^13^, the escape room used in this study was structured so that it could also be solved individually. Larger team sizes risked reducing the number of tasks per participant, thereby limiting individual learning opportunities, whereas smaller groups promoted interaction and collaborative learning. Based on these considerations, the group size was set to three to four participants.

#### Group B (lecture group)

Participants attended a 30-minute interactive lecture delivered by a family physician.

#### Group C (control group)

No educational intervention was provided; only the questionnaires were administered.

Attendance was recorded using student identification numbers, and participation on multiple days was prohibited. Participants who attended the September 24 session were explicitly instructed not to disclose the session content to others. Because Group A’s session took place during the lunch break, light snacks were provided; to avoid potential bias in responses, the same snacks were also provided to Group B. Participants who completed the three-month follow-up questionnaire received a ¥2,000 Amazon gift card as an honorarium.

For the escape room intervention, *Escape Room to Understand Illness Nazopital Vol. 1: Phantom Thief Moriwarl and the Secret Vaccine*, a puzzle-based educational game designed to promote understanding of disease, was used. This escape room was developed by Dr.GAMES, a general incorporated association with which one of the co-investigators (Kondo) is affiliated, and it used a LINE chatbot integrated with paper-based materials. The game was originally designed for elementary and junior high school students, allowing them to take the materials home and solve the puzzles with the support of adults. In the present study, participants solved ciphers and puzzles labeled A-D in “Chapter 1A” (Figure S1) while referring to supplementary information in “Chapter 1B” (Figure S2). They entered their answers into the LINE chatbot to automatically advance the storyline (Figure S3). On clearing Chap. 1, participants answered four HPV vaccine-related quiz questions via the LINE chatbot (Figure S4). Incorrect responses prompted the same question to reappear until a correct answer was selected; no explanatory feedback was provided within the chatbot. These questions corresponded to four of the seven knowledge test items administered before the intervention, immediately after, and at the three-month follow-up. Correct responses allowed participants to progress to “Chapter 2,” (Figure S5) where they completed a crossword puzzle and followed the given instructions to derive the final solution. On average, the activity could be completed in approximately 30 min, and its content was designed to be accessible to both children and adults without requiring any specialized prior knowledge.

The HPV vaccine-related knowledge items incorporated into the escape room were selected based on literature on cervical cancer^14–17^, and they were finalized through a focus group discussion involving pediatricians, obstetrician-gynecologists, and public health specialists. For the intervention, the escape room was conducted in a university classroom, with participants working in groups of three to four under a 30-minute time limit. No additional briefing, debriefing, or supplementary lectures were provided before or after the activity. Three faculty members supervised the session: one served as the game master, and the other two assisted with tasks such as distributing the game booklets. During the escape room activity, no instructional input or lectures were provided by faculty; rather, students engaged in the escape room autonomously under faculty supervision.

The lecture for the traditional class group was delivered by a general medicine physician (ninth year post-graduation, board-certified in general medicine and family medicine) who was not involved in this study. In addition to clinical practice, this physician was involved in the education of medical students and residents and was well-versed in medical education. The lecture was conducted in an interactive format, incorporating an audience response system.

The lecturer was specifically instructed to:

1. Base the lecture content on the HPV vaccine educational material distributed to the escape room group (Figure S2).
2. Incorporate the same four quiz questions that were used in the escape room, and immediately provide feedback on quiz answers.

The lecture lasted approximately 30 min, matching the escape room session in duration to ensure educational equivalence across groups.

In the control group, only the baseline questionnaire was administered to students who gathered in the classroom on the study day.

## Outcome measures

Participants in the escape room and lecture groups completed questionnaires at three time points: pre-class (T0), immediately post-class (T1), and three months post-class (T2). Participants in the control group completed questionnaires at two time points (T0 and T2) and did not complete the immediate post-class survey.

In the pre-class survey, the following items were assessed:

1. **Demographic characteristics**: student identification number, email address, age, sex, academic year, and faculty.
2. **Background information on HPV vaccination**: history of contraindications to HPV vaccination (including medical reasons precluding vaccination), awareness of the HPV vaccine, history of HPV vaccination, history of cervical cancer screening, and whether participants would recommend HPV vaccination to family or friends. Participants who had received at least one dose were categorized as having a history of HPV vaccination, since HPV vaccination is administered as a multi-dose series. For participants who were unvaccinated, vaccination intention was also assessed. Vaccination intention was assessed only in participants who had never received the HPV vaccine (0 doses). Vaccination intention and uptake were evaluated in participants who were unvaccinated at baseline (0 doses), thereby minimizing confounding by baseline vaccination history.
3. **Knowledge test**: a seven-item multiple-choice test assessing basic knowledge of cervical cancer and HPV vaccination. The content of the knowledge test is provided in Table 1. All items were presented in single-choice format with four answer options consistent with the design of the LINE chatbot used in the escape room intervention. Questions 1–4 were also incorporated directly into both the escape room and lecture-based interventions, whereas information relevant to questions 5–7 was included in the handout (Figure S2). The handout was distributed to both the escape room and lecture groups to ensure content equivalence. The seven questions were developed with supervision and feedback from a multidisciplinary panel (four obstetrician-gynecologists, two pediatricians, and three public health specialists; all physicians had > 10 years of clinical experience) to ensure content relevance and appropriateness. At the time of study design, no HPV knowledge scale with established reliability and validity was available in Japanese. Although validated international instruments exist, they are substantially longer and would require translation/cultural adaptation; incorporating a larger number of factual items would also have increased respondent burden and risked disrupting the flow and enjoyment of the escape room activity. Therefore, a brief seven-item test aligned with the core learning objectives of both interventions was used.

**Table 1:** Knowledge Test Items on HPV Vaccine and Cervical Cancer.

No.	Question	Response Options	Answer
1	In which age group is cervical cancer most common?	(1) 20–40 years (2) 50–60 years (3) 70–80 years (4) Do not know	1. 20–40 years
2	What is the primary cause of cervical cancer?	(1) Viral infection (2) Overeating (3) Alcohol consumption (4) Do not know	1. Viral infection
3	By what percentage can HPV vaccination reduce the risk of developing cervical cancer?	(1) 10–20% (2) 30–50% (3) 60–90% (4) Do not know	3. 60–90%
4	Who is the target population for routine HPV vaccination in Japan?	(1) Grades 6–10 (equivalent to ages 12–16) (2) Grades 10–12 (equivalent to ages 16–18) (3) During university years (4) Do not know	1. Grades 6–10 (equivalent to ages 12–16)
5	Until when can women born between April 2, 1997, and April 1, 2008, who have not previously received HPV vaccination, receive it free of charge in Japan?	(1) Until December 2024 (2) Until March 2025 (3) Until March 2026 (4) Do not know	2. Until March 2025
6	What is the primary route of HPV transmission?	(1) Through blood (2) Through droplets such as coughing or sneezing (3) Through sexual contact (4) Do not know	3. Through sexual contact
7	Approximately what percentage of sexually active individuals are estimated to be infected with HPV?	(1) 10–20% (2) 30–40% (3) 50–80% (4) Do not know	3. 50–80%

Immediately after the class, participants in the escape room and lecture groups completed a post-class survey assessing satisfaction, perceived (self-reported) comprehension, intention to receive the HPV vaccine, and HPV-related knowledge. Satisfaction and perceived comprehension were assessed using two single-item statements: “I am overall satisfied with the class” and “I understood the class well.” Responses were rated on 10-point Likert scales (1 = not at all, 10 = extremely). Intention to receive the HPV vaccine was assessed only in participants who had never received the HPV vaccine (0 doses) using a 7-point Likert scale ranging from “strongly agree” to “strongly disagree.” Participants also repeated the same seven-item knowledge test administered before the class.

In the three-month follow-up survey, participants were asked whether they had received at least one dose of the HPV vaccine since baseline (vaccination initiation). Vaccination status was self-reported and not verified against vaccination records or immunization registries. Participants who reported remaining unvaccinated were asked about their intention to receive HPV vaccination using the same 7-point Likert scale. The seven-item knowledge test identical to that used in the pre- and post-class surveys was also administered at this time point.

### Data analysis

All statistical analyses were performed using JMP Pro, version 18 (JMP Statistical Discovery LLC, Cary, NC, USA). Continuous variables are presented as median with interquartile range (IQR) values, and categorical variables as percent (%). Categorical data such as participant characteristics were compared using the chi-squared test. When the overall test indicated a significant between-group difference, post hoc pairwise comparisons were conducted, and p-values were adjusted using the Holm-Bonferroni method to account for multiple comparisons. For continuous variables, normality was assessed, and paired *t*-tests were used as appropriate. A two-sided *P* value < 0.05 was considered significant. Because there were significant between-group differences in baseline knowledge test scores, analysis of covariance (ANCOVA) was conducted with the pre-class test score as a covariate and instructional method as a fixed factor to compare the intervention and control groups. Cases with missing data were analyzed using available (non-missing) data only.

### Open-ended questionnaire items

To capture higher-order cognitive and affective outcomes that are difficult to assess using closed-ended measures alone, open-ended items were included in the post-class questionnaire. These items were administered only to the escape room group (Group A) immediately after the intervention to explore perceived advantages, disadvantages, and further learning needs. The wording of the open-ended prompts was refined through iterative discussions between two investigators (KK and KI).

Students in the escape room group were presented with the following open-ended questions in the post-class questionnaire:

- *What do you think are the advantages of puzzle-solving for learning about the HPV vaccine? Why do you think so?*
- *What do you think are the disadvantages of puzzle-solving for learning about the HPV vaccine? Why do you think so?*
- *After completing the puzzle-solving activity*,* what further aspects of the HPV vaccine would you like to learn about?*
- *Please provide any additional comments or opinions regarding the puzzle-solving activity.*

Qualitative data were analyzed using content analysis^18,19^. As the starting point of the analysis, a preliminary coding framework was developed based on Fink’s taxonomy. Fink’s taxonomy is an educational framework comprising six dimensions of the learning process: *learning how to learn*,* caring*,* human dimension*,* integration*,* application*, and *foundational knowledge* (Table S1). Unlike Bloom’s taxonomy, another widely used educational framework, Fink’s taxonomy explicitly incorporates social and affective elements^20^. Given that the escape room used in this study was designed to foster emotional engagement and collaboration among participants, its potential impact was assumed to extend beyond knowledge acquisition to include values and behavioral change. Therefore, Fink’s taxonomy was adopted as the analytical framework.

Two researchers (KK and TM) independently read all responses in full and performed coding. To ensure rigor, researcher triangulation was applied: KK and TM conducted the analysis and reached consensus through discussion. Categories and subcategories emerging from the data were subsequently reviewed and refined through regular discussions with KI, a researcher experienced in qualitative methods, to enhance credibility. This qualitative study was conducted in accordance with the Consolidated Criteria for Reporting Qualitative Research (COREQ)^21^. Analytical categories were organized according to the six dimensions of Fink’s taxonomy: *learning how to learn*,* caring*,* human dimension*,* integration*,* application*, and *foundational knowledge*^22^. Following open coding, similar codes were grouped into subcategories. Each concept within the six dimensions of Fink’s taxonomy was analyzed, and the frequency of units coded to each dimension was calculated. In addition, similar codes were grouped into overarching themes, which were then mapped to the corresponding dimensions of Fink’s taxonomy.

### Content analysis of open-ended questionnaire responses

To contextualize the quantitative outcomes and capture learners’ reflections that may not be fully assessed using closed-ended measures alone, open-ended items were included in the post-class questionnaire for the escape room group only. Free-text responses were analyzed using content analysis and summarized as category frequencies (with illustrative quotes) based on Fink’s taxonomy.

## Results

### Participants’ characteristics

**Fig. 1:**
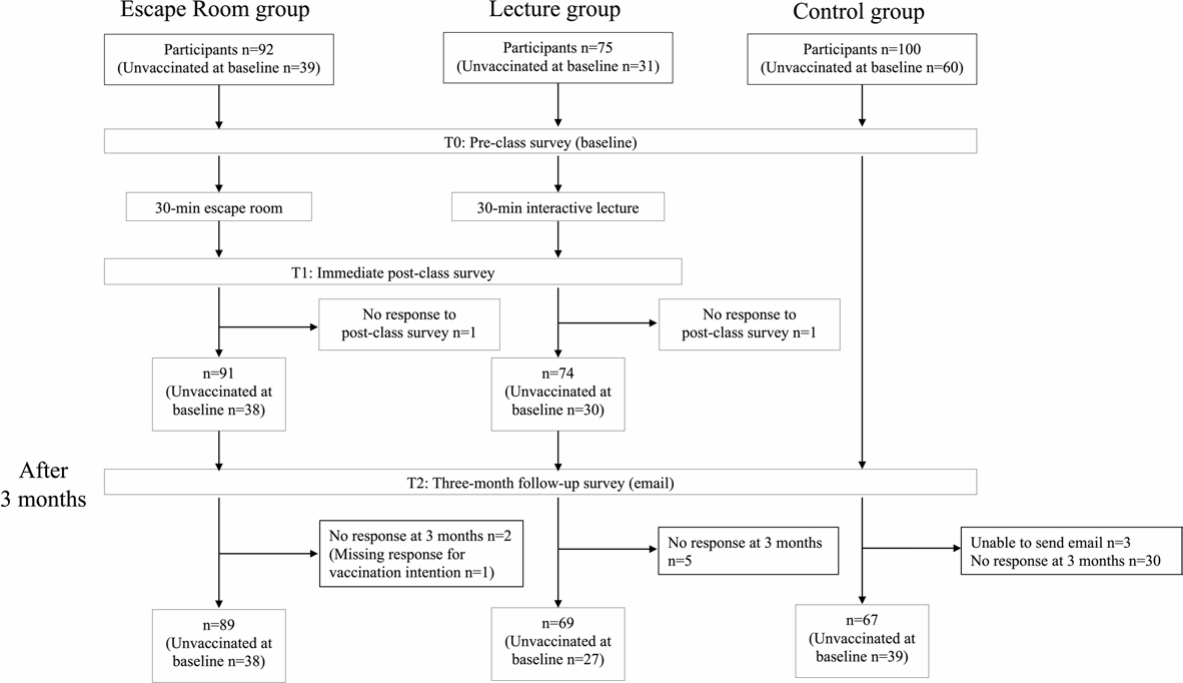
Participant flow and timing of questionnaire administration.

A total of 267 students participated in the study: 92 in the escape room group, 75 in the lecture group, and 100 in the control group. No participants were excluded. At the three-month follow-up, responses were obtained from 89 students in the escape room group, 69 in the lecture group, and 67 in the control group (Fig. 1). At baseline, participants’ characteristics differed across groups; notably, the control group reported a lower history of HPV vaccination than the two intervention groups (Table 2).

**Table 2:** Participants’ baseline characteristics.

Parameter	Total (*n* = 267)	Escape Room (*n* = 92)	Lecture (*n* = 75)	Control (*n* = 100)	*p* value
Female **[This row is superfluous**,** since all participants had to be female.]**	267	92	75	100	
Age, y, mean ± SD	19.8 ± 1.6	20.2 ± 1.3	20.3 ± 1.7	19.1 ± 1.4	< 0.001
Awareness of HPV vaccine prior to the study, n (%)	238 (89.1)	81 (88.0)	65 (86.7)	92 (92.0)	0.478
History of HPV vaccination, n (%)	137 (51.3)	53 (57.6)	44 (58.7)	40 (40.0)	0.017
History of cervical cancer screening, n (%)	45 (16.9)	16 (17.4)	10 (13.3)	19 (19.0)	0.603

### Satisfaction and comprehension of the class

Immediately after the class, the satisfaction scores were 8.20 ± 2.16 in the escape room group and 9.11 ± 1.21 in the lecture group, with the lecture group showing significantly higher satisfaction (*p* = 0.002). The perceived (self-reported) comprehension scores were 8.07 ± 2.12 in the escape room group and 9.46 ± 0.95 in the lecture group, with the lecture group showing significantly higher self-reported comprehension (*p* < 0.001) (Table 3).

**Table 3:** Satisfaction and perceived (self-reported) comprehension immediately after the class (intervention groups only).

Parameter	Total (*n* = 267)	Escape Room (*n* = 92)	Lecture (*n* = 75)	Control (*n* = 100)	*p* value
Class satisfaction (10-point scale), mean ± SD	-	8.20 ± 2.16	9.11 ± 1.21	-	0.002
Class comprehension (10-point scale), mean ± SD	-	8.07 ± 2.12	9.46 ± 0.95	-	< 0.001

### Knowledge test scores after the intervention

Immediately after the class, the number of correct answers on the knowledge test was significantly higher in the lecture group than in the escape room group. However, at the three-month follow-up, both the escape room and lecture groups had significantly higher scores than the control group, with no significant difference between the escape room and lecture groups (Table 4). To further explore potential mechanisms, item-level analyses of the knowledge test were conducted immediately after the intervention. For each item, an ANCOVA model was fitted with the post-intervention item score as the dependent variable and the corresponding pre-intervention item score as a covariate. Adjusted correct response rates did not differ between groups for items 1–4 (all *p* ≥ 0.121). In contrast, the lecture group showed higher adjusted correct response rates for item 5 (mean difference [escape room − lecture] = − 0.200, 95% CI − 0.308 to − 0.092; *p* < 0.001) and item 7 (− 0.172, 95% CI − 0.290 to − 0.053; *p* = 0.005), with a marginal difference for item 6 (− 0.063, 95% CI − 0.128 to 0.001; *p* = 0.053) (Table S2).

**Table 4:** HPV vaccine knowledge test scores across time (ANCOVA-adjusted).

Parameter	Total (*n* = 267)	Escape Room (*n* = 92)	Lecture (*n* = 75)	Control (*n* = 100)	*p* value
Pre-intervention to post-intervention	-	6.328 ± 0.101	6.751 ± 0.116	-	0.008
Pre-intervention to 3-month follow-up	-	5.763 ± 0.117	5.900 ± 0.134	-	0.454
Pre-intervention to 3-month follow-up	-	5.565 ± 0.134	-	4.116 ± 0.155	< 0.001
Pre-intervention to 3-month follow-up	-	-	5.814 ± 0.147	4.326 ± 0.149	< 0.001

Values are adjusted means (± SE) from ANCOVA controlling for baseline knowledge test scores.

### Intention to receive HPV vaccination

Among participants who had not previously received the HPV vaccine (*n* = 130), the mean intention score at baseline did not differ significantly among the groups (escape room: 4.0 ± 1.5; lecture: 4.8 ± 1.6; control: 4.3 ± 1.3; *p* = 0.078). Immediately after the intervention, however, vaccination intention was significantly higher in the lecture group (5.7 ± 1.3) than in the escape room (4.3 ± 1.7) and control groups (4.3 ± 1.3; *p* < 0.001). The mean change in intention from pre- to post-intervention was also greater in the lecture group (+ 0.967) than in the escape room group (+ 0.421; *p* = 0.027).

At the three-month follow-up, the analysis restricted to participants who remained unvaccinated (*n* = 76) showed no significant between-group differences in mean intention scores (escape room: 4.6 ± 1.2; lecture: 4.6 ± 1.7; control: 3.9 ± 1.6; *p* = 0.162). However, the mean change in intention from baseline to three months was significantly greater in the escape room group (+ 0.897) than in the control group (–0.121; *p* = 0.025) (Table 5).

**Table 5:** Intention to receive HPV vaccination over time in unvaccinated participants (0 doses).

Parameter	Total	Escape Room	Lecture	Control	*p* value
**Pre-intervention** (7-point Likert scale)	*n* = 130	*n* = 39	*n* = 31	*n* = 60	
Intention to receive HPV vaccine, mean ± SD	4.3 ± 1.5	4.0 ± 1.5	4.8 ± 1.6	4.3 ± 1.3	0.078
**Post-intervention** (7-point Likert scale)	*n* = 68	*n* = 38	*n* = 30		
Intention to receive HPV vaccine, mean ± SD	4.9 ± 1.7	4.3 ± 1.7	5.7 ± 1.3	-	< 0.001
Change in intention (Pre to Post), mean difference	-	0.421	0.967	-	0.027
**Three-month follow-up** (7-point Likert scale, unvaccinated at 3 months only)	*n* = 76	*n* = 29*	*n* = 14	*n* = 33	
Intention to receive HPV vaccine, mean ± SD 3-month follow-up	4.3 ± 1.5	4.6 ± 1.2	4.6 ± 1.7	3.9 ± 1.6	0.162
Change in intention (Pre to 3-month follow-up), mean difference	-	0.897	0.286	-0.121	0.025

* One participant had a missing response for vaccination intention at the three-month follow-up (*n* = 1).

### HPV vaccination initiation at three-month follow-up

At the three-month follow-up, in participants unvaccinated at baseline (0 doses; *n* = 104), the proportion initiating HPV vaccination (≥ 1 dose) differed significantly across the three groups (escape room: 8/38 [21.1%], lecture: 13/27 [48.2%], control: 6/39 [15.4%]; overall *p* = 0.007) (Table 6). Post hoc pairwise comparisons with Holm-Bonferroni adjustment indicated that vaccination initiation was significantly higher in the lecture group than in the escape room group (*p*_adj = 0.043) and the control group (*p*_adj = 0.012), whereas there was no significant difference between the escape room and control groups (*p*_adj = 0.519).

**Table 6:** Self-reported HPV vaccination initiation at 3 months in participants unvaccinated at baseline (0 doses).

Parameter	Total (*n* = 104)	Escape Room (*n* = 38)	Lecture (*n* = 27)	Control (*n* = 39)	*p* value*
HPV vaccination initiation by 3 months (≥ 1 dose), n (%)	27	8 (21.1)	13 (48.2)	6 (15.4)	0.007

* Overall comparison across three groups using the chi-squared test.

† Post hoc pairwise comparisons were performed using the chi-squared test with Holm-Bonferroni adjustment: Lecture vs. Escape Room, *p*adj = 0.043; Lecture vs. Control, *p*adj = 0.012; Escape Room vs. Control, *p*adj = 0.519.

‡ Vaccination initiation was self-reported and was not verified using vaccination records or immunization registries.

### Content analysis

Immediately after the escape room session, an open-ended questionnaire survey was administered, with responses from 92 participants included in the analysis. Analytical categories were developed according to Fink’s taxonomy. Following open coding, similar codes were grouped into subcategories and then categorized into higher-order themes, which were subsequently mapped onto the six dimensions of Fink’s taxonomy. After analyzing all responses, data saturation was confirmed.

A total of 190 codes were extracted from the free-text responses. From these, 22 subcategories corresponding to six themes aligned with the six dimensions of Fink’s taxonomy were identified: *Learning how to learn* (15), *Caring* (84), *Human dimension* (15), *Integration* (15), *Application* (21), and *Foundational knowledge* (40). Of them, the most frequently coded dimension was *Caring*. Representative themes, subcategories, and illustrative quotes are shown in Table 7.

**Table 7:** Themes, subcategories, and illustrative quotes identified from content analysis.

Theme (Number of theme)	Subcategory (Number of subcategory)	Illustrative Quotes
Caring (84)	Enhancement of Engagement (63)	Though the term “lesson” often evokes a sense of reluctance, the escape room activity provided an enjoyable and effective learning experience. (ID = 73)
	Self-regulated learning attitude (10)	I find it difficult to stay engaged during lectures where I only listen passively, but actively thinking and enjoying the process made it easier for me to absorb the information. (ID = 3)
	Enhancement of Learning Motivation (8)	The presence of interactive elements increased my motivation to learn. (ID = 52)
	Facilitation of Learning Difficult Topics (2)	Being taught as part of sex education makes me feel defensive, whereas the escape room activity allowed me to engage more comfortably and openly. (ID = 12)
	Retained Engagement in Learning (1)	Compared to regular lessons, it was less likely to become boring. (ID = 11)
Foundational Knowledge (45)	Acquiring Knowledge (29)	Engaging in the escape room activity naturally involved reading explanations about the HPV vaccine, facilitating knowledge acquisition. (ID = 17)
	Facilitation of Understanding (8)	Having the answers consist of key terms related to the HPV vaccine made it easier to learn and understand the content. (ID = 70)
	Knowledge Retention (8)	I perceived that acquiring knowledge through the escape room activity was more effective in promoting knowledge retention than studying on my own. (ID = 2)
Application (19)	Encouragement of Reflective Thinking (12)	By thinking deeply and formulating answers independently, I was able to develop a deeper understanding of the final completed answer. (ID = 5)
	Facilitation of Information Processing (5)	Vaccine information was naturally presented, allowing me to absorb it effortlessly. (ID = 35)
	Linking Knowledge to Practice (1)	The answers were directly related to the topic. (ID = 18)
	Skill of Identifying Key Information (1)	Key terms were learned naturally through the escape room activity. (ID = 19)
Learning How to Learn (15)	Lowering Barriers to Learning (12)	Learning became enjoyable and approachable through conversations with friends. (ID = 66)
	Establishment of Psychological Safety (1)	Structuring the lesson as an escape room activity lowered the psychological barrier to learning about the HPV vaccine compared with a conventional lesson. (ID = 32)
	Meta-cognitive Learning Style (1)	The encouragement to get vaccinated was conveyed from the viewpoint of a third-party character within the game. (ID = 84)
	Visual Learning Support (1)	As vaccine-related terms were essential to solving the puzzles, participants inevitably encountered the information at least once. (ID = 80)
Human Dimension (15)	Collaborative learning with peers (11)	It provided an opportunity to experience the joy of collaborating with friends. (ID = 74)
	Team-Based Communication (4)	The activity facilitated communication and enabled discussions about the HPV vaccine. (ID = 36)
Integration (7)	Comprehensive Information Processing (6)	Reading through all the materials during the escape room activity facilitated a deeper absorption of the content. (ID = 8)
	Visualization of Thought Processes (1)	It facilitated the conscious recognition of key terms. (ID = 56)

## Discussion

This study evaluated the effectiveness of an educational intervention using an escape room, a form of experiential learning, as a novel approach for HPV vaccine education, in comparison with a lecture group and a non-intervention control group. In addition, a qualitative evaluation was conducted specifically in the escape room group to explore higher-order cognitive and affective learning outcomes, such as emotional engagement and learner autonomy, which are difficult to capture solely through quantitative measures.

Quantitative findings demonstrated that both the escape room group and lecture group achieved significantly higher correct response rates on HPV knowledge tests than the control group, and these effects were maintained at the three-month follow-up. Notably, immediately after the intervention, the lecture group showed greater improvements in knowledge scores than the escape room group; however, at three months, knowledge retention was comparable between the two groups. This suggests that an escape room approach, which can be implemented with limited time and human resources, may represent an effective strategy for vaccine education of university students. Item-level analyses immediately after the intervention suggested that this short-term difference was driven primarily by items requiring information present only in the distributed handout (items 5–7), whereas items 1–4 showed no between-group differences (Table S2). One possible explanation is that the interactive lecture, which included instructor feedback on quiz items, may have provided learners with cues about which content was important and where to locate relevant information in the handout, thereby facilitating more efficient searching and integration. Conversely, the escape room activity may impose higher cognitive load due to puzzle-solving and time pressure, leaving less cognitive capacity to return to and process content exclusive to the handout.

Qualitative findings from the escape room group showed responses corresponding to all six dimensions of Fink’s taxonomy; of them, the most frequently coded dimension was *Caring*, suggesting that the escape room functioned not merely as a means of knowledge transmission, but also as an educational environment that evoked emotional engagement and intrinsic motivation through a game-based format. Within the subcategories, “Enhancement of Engagement” was the most frequently identified, underscoring how the entertainment aspects of the game fostered educational involvement. Other frequent subcategories included “Acquiring Knowledge,” “Lowering Barriers to Learning,” “Collaborative Learning with Peers,” and “Self-Regulated Learning Attitude,” indicating that the escape room created a learning environment that facilitated accessibility, dialogue, and autonomous learning. These findings suggest that the process of active thinking and problem-solving within the escape room promoted personal relevance and engagement with vaccination. Moreover, many participants reported that independently reflecting during the activity deepened their understanding, an educational benefit that is less likely to be achieved in traditional didactic lectures and should be emphasized as a key advantage of experiential learning. Such approaches are also important in fostering collaborative decision-making and information-processing skills, which are critical in the healthcare field, and may ultimately be applicable to professional education.

Regarding actual vaccination behavior, the lecture group demonstrated a significant increase in HPV vaccination uptake, whereas the escape room group did not differ significantly from the control group. This finding aligns with prior research, including the systematic review by Ou et al., which concluded that, though game-based interventions are effective in modifying knowledge and attitudes, their impact on actual behavioral change is limited^7^. This “intention-behavior gap”^23^ is well recognized in behavioral science, since actual behavior is influenced not only by information provision, but also by multifactorial determinants such as accessibility, social support, and the removal of economic or psychological barriers. In the present study, the lecture group’s in-person interaction with a physician, which allowed participants to have their questions and concerns addressed, may have contributed to behavioral change, whereas this component was lacking in the escape room group. In addition, the lecture was delivered by a physician, a trusted health professional and authoritative source of vaccine information, which may have amplified the intervention effects beyond the instructional format itself. Given the well-established influence of healthcare providers’ recommendations on vaccination intentions and uptake^24,25^, the higher satisfaction, perceived comprehension, knowledge gains, intention, and uptake observed in the lecture group may have partially reflected this so-called “messenger effect.” Future studies should control for instructor effects and opportunities for real-time Q&A (e.g., using the same facilitator across conditions or adding a standardized physician-led debriefing to the escape room) to disentangle the effect of the educational format from the effect of the messenger. These results indicate that the escape room alone may be insufficient to achieve behavioral change, and that future educational strategies should consider hybrid approaches, combining the escape room with debriefing sessions or professional dialogue, to bridge the gap between knowledge and behavior.

The escape room used in this study was designed using paper-based materials in conjunction with a LINE chatbot, enabling implementation by a single instructor. Importantly, no supplementary elements such as pre- or post-intervention lectures or debriefing were included, yet the escape room alone was sufficient to demonstrate educational effectiveness. In Japan, multiple escape room formats exist, including the “Room” Type commonly used in Western countries, as well as “Hall”, “Stadium”, and “Field” Types^26^. Although the escape room used in the present study was originally designed for individual or small-group completion at home, it was adapted here to a Hall Type format for group use. This adaptation highlights a practical advantage of the escape room, namely its feasibility in educational settings with limited personnel and resources. By lowering the logistical barriers traditionally associated with the escape room, such as venue, equipment, and staffing requirements, this approach may facilitate wider adoption in educational contexts.

This study has several limitations. First, the participants were students from a single university with a relatively high baseline HPV vaccination rate. Therefore, compared with the general student population, their hesitancy toward vaccination may have been lower, potentially leading to an underestimation of the intervention’s true effect. Second, the control group experienced a relatively high dropout rate at the three-month follow-up. This may have been attributable to decreased motivation and diminished interest, given that the control group did not receive any educational intervention. Such attrition could have influenced the evaluation of long-term outcomes such as vaccination uptake and knowledge retention, thereby reducing the representativeness of the control group in three-arm comparisons. Future studies should consider strategies to maintain engagement in non-intervention groups, for example by providing an unrelated educational session, to minimize dropout. Third, because participants selected sessions based on date/time availability, group allocation was not randomized, and baseline non-equivalence may have occurred (e.g., differences in prior HPV vaccination history). This selection bias may have affected between-group comparisons. Consistent with this concern, there were baseline differences in knowledge levels and vaccination intention across groups, limiting the internal validity of between-group comparisons. Future research should adopt stratified randomization or matching methods to address this issue. Fourth, several outcome measures, including HPV vaccination initiation, satisfaction, comprehension, and vaccination intention, were self-reported; vaccination initiation was not verified using vaccination records or immunization registries, and thus misclassification due to recall or social desirability bias is possible. Fifth, the knowledge test consisted of only seven items. In addition, HPV knowledge was assessed using a study-specific short test, and its psychometric properties have not been formally established. Future studies should consider using validated Japanese instruments (e.g., the Japan HPV Knowledge Scale^26^ and/or conducting formal reliability and validity testing of brief knowledge measures tailored for game-based learning interventions. A larger number of items might have affected the accuracy of the knowledge assessment. However, considering the constraints of testing time and participant burden, adding further questions was not feasible. Moreover, the test items were derived from the educational material provided (Figure S2), which limited the scope of questions. Increasing the number of questions within the escape room could also have diminished the enjoyment and immersive nature of the game, thereby undermining its educational effect. Thus, the decision to limit the number of questions was considered appropriate. Sixth, the follow-up period was limited to three months, which was insufficient to assess long-term knowledge retention and sustained changes in vaccination behavior. In addition, because HPV vaccination is administered as a multi-dose series, and the follow-up period was limited to three months, whether participants who initiated vaccination subsequently completed the recommended dosing schedule could not be determined. Therefore, since the present findings reflect vaccination initiation rather than series completion, longer-term evaluations of six months to one year are warranted. Seventh, it was difficult to isolate the sustained effects observed at the three-month follow-up in any of the study conditions. Participants in all groups may have been exposed to other information sources during the post-intervention period, such as media, peers, or the university. Therefore, the follow-up outcomes cannot be attributed with certainty to the initial assignment alone. Future studies should incorporate designs that reduce contamination and enable clearer counterfactual comparisons. Eighth, although this study assessed the educational effect of the escape room alone, it is generally recommended that debriefing sessions or supplementary classes be incorporated as part of the reflective process described in Kolb’s experiential learning cycle^12^. Due to time constraints, these elements were not implemented in the present study; however, their inclusion may further enhance the educational impact of the intervention. In addition, qualitative data were collected only from the escape room group; therefore, qualitative comparisons between the escape room and lecture interventions were not possible. Future studies should collect qualitative data from both intervention groups to directly compare learning processes and higher-order outcomes. Finally, the qualitative data collected in this study were limited to open-ended survey responses. This design did not allow for a detailed exploration of which specific elements of the escape room (e.g., storyline, interactivity, or media format) contributed to the educational outcomes. Future research should use more in-depth qualitative methods, such as semi-structured interviews or focus groups, to elucidate these aspects.

This study represents the first attempt in Japan to demonstrate the potential of the escape room for vaccine education, providing multifaceted evidence of its value and practicality as a form of experiential learning. The findings underscore the utility of the escape room as an innovative educational strategy in public health and mark an important step toward its broader application in health education.

## Conclusion

This study evaluated the effectiveness of an escape room approach for HPV vaccine education and demonstrated that it was comparable to traditional lectures in improving knowledge and fostering active learning. In particular, a hybrid escape room format combining paper-based materials with a LINE chatbot appears to be a promising educational strategy that can be implemented at low cost with small groups. However, self-reported vaccination initiation was higher in the lecture group, indicating that additional interventions may be required to bridge the gap between knowledge and behavior. Future research should compare different escape room formats, assess long-term outcomes, and expand the target population to include middle and high school students, as well as young adults. Moreover, escape room-based approaches may be applied to other health domains, such as influenza, sexually transmitted infections, and lifestyle-related diseases. As a novel educational strategy, the escape room approach has the potential to promote health behaviors in younger populations and to make a substantial contribution to public health education.

## Supplementary Information

Below is the link to the electronic supplementary material.

Supplementary Material 1

## Data Availability

The datasets generated and analyzed during the current study are available from the corresponding author on reasonable request.
