# Supplementary material for: Escape room-based learning for HPV vaccine education in Japanese female university students: a controlled, three-group study
Source: Sci Rep. 2026 Apr 18;16:17987. doi: 10.1038/s41598-026-48126-w (PMC13249944; doi:10.1038/s41598-026-48126-w)
Supplement: Supplementary file 1 — Supplementary Material 1 [file 41598_2026_48126_MOESM1_ESM.pdf]

Supplementary Information

Figure S1. *Escape Room to Understand Illness Nazopital Vol. 1: Phantom Thief Moriwarl and the Secret Vaccine Chapter 1A (front/back)*

Annotation: Though the original version used in the study was in Japanese, an English translation of the relevant components is presented here for publication.

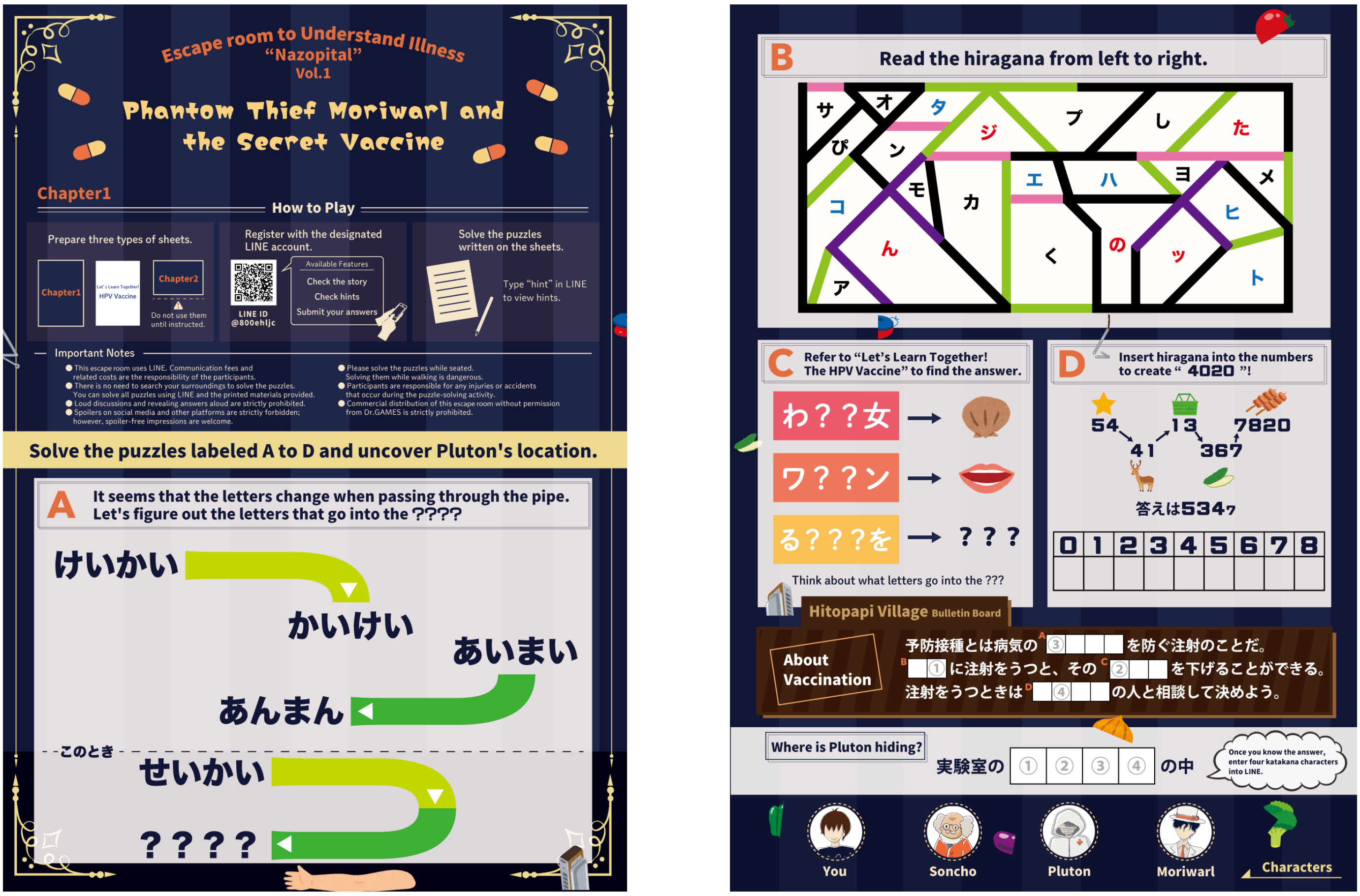

Figure S2. *Escape Room to Understand Illness Nazopital Vol. 1: Phantom Thief Moriwarl and the Secret Vaccine, Chapter 1B* - educational material on HPV vaccine knowledge

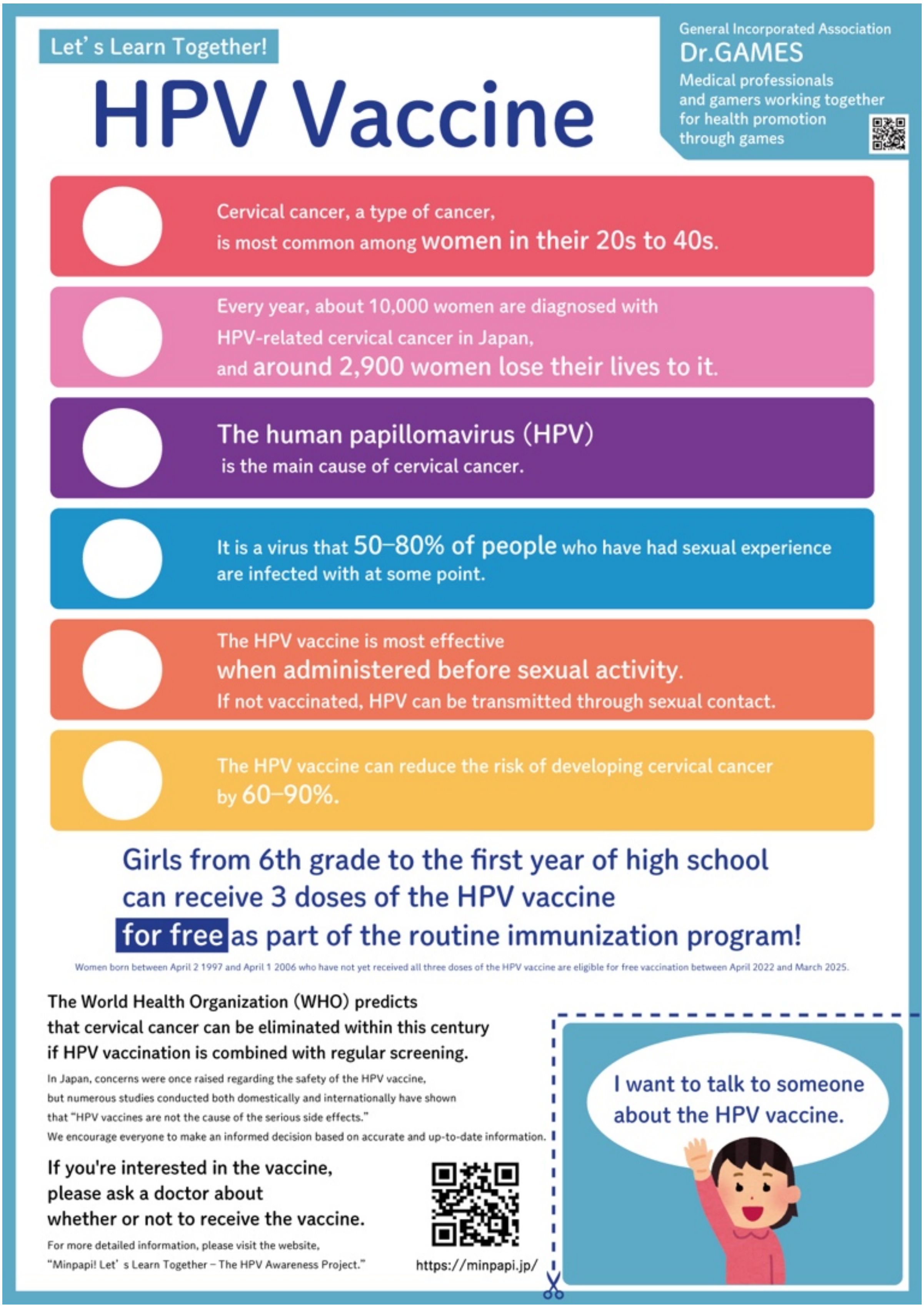

Figure S3. *Escape Room to Understand Illness Nazopital Vol. 1: Phantom Thief Moriwarl and the Secret Vaccine,* LINE chatbot game interface (story section)

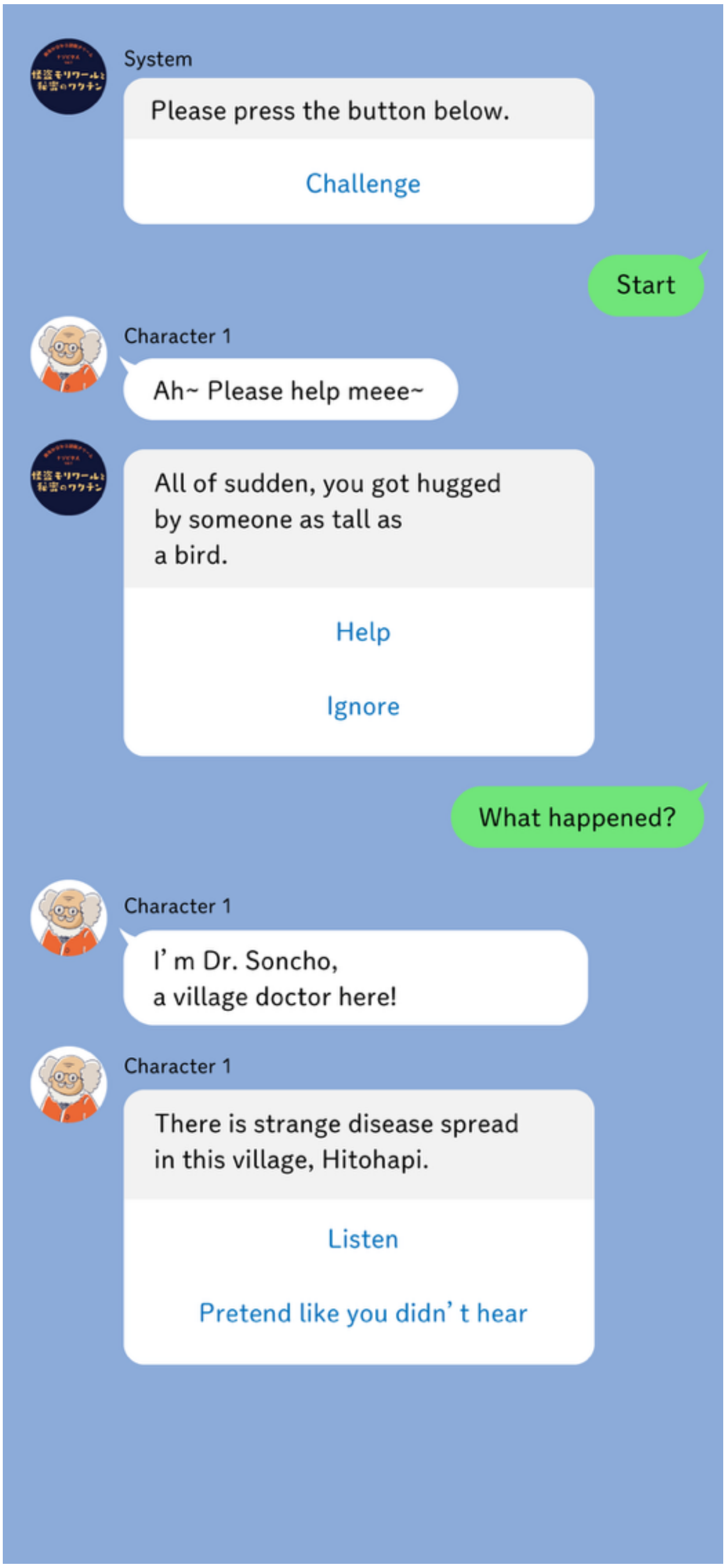

Figure S4. *Escape Room to Understand Illness Nazopital Vol. 1: Phantom Thief Moriwarl and the Secret Vaccine,* LINE chatbot game interface (quiz section)

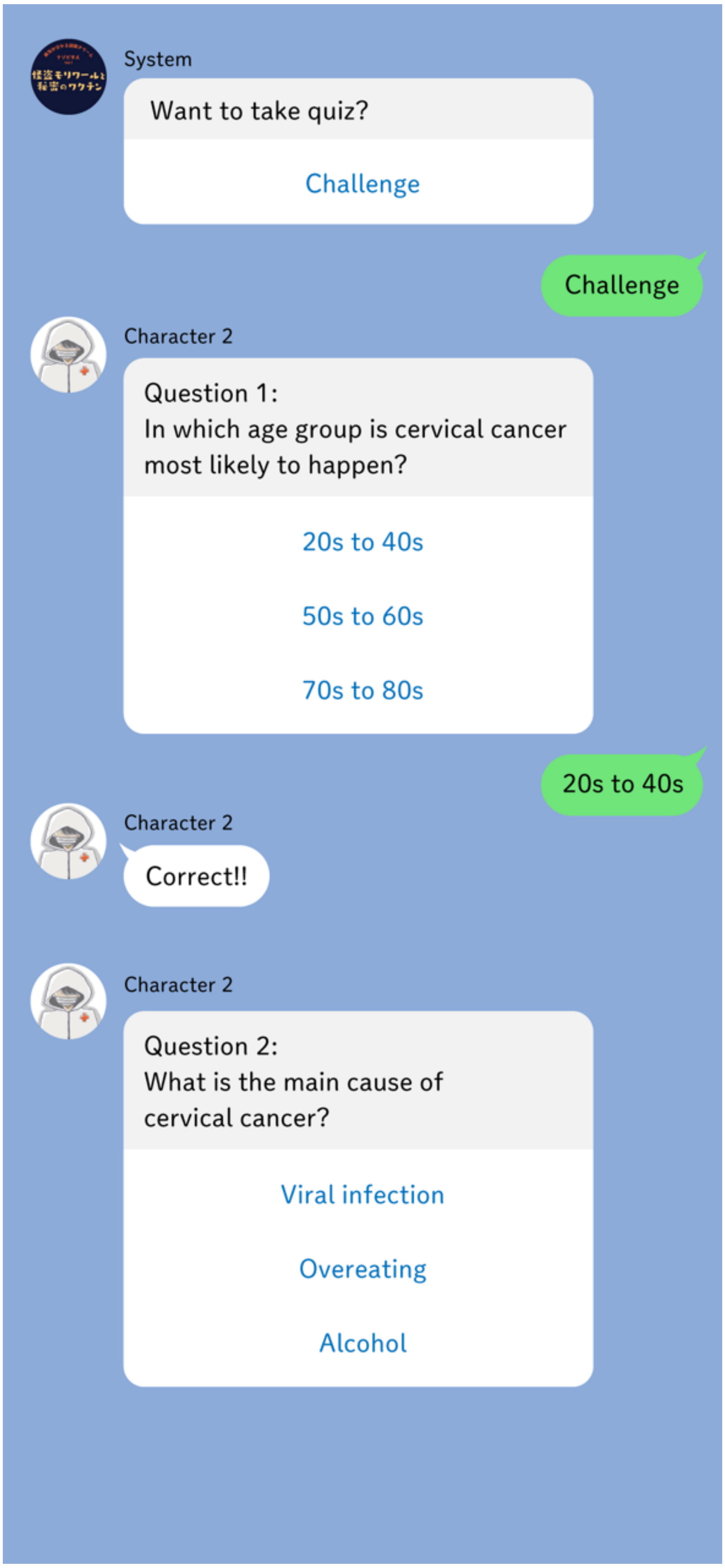

Figure S5. *Escape Room to Understand Illness Nazopital Vol. 1: Phantom Thief Moriwarl and the Secret Vaccine,* Chapter 2 (front/back)

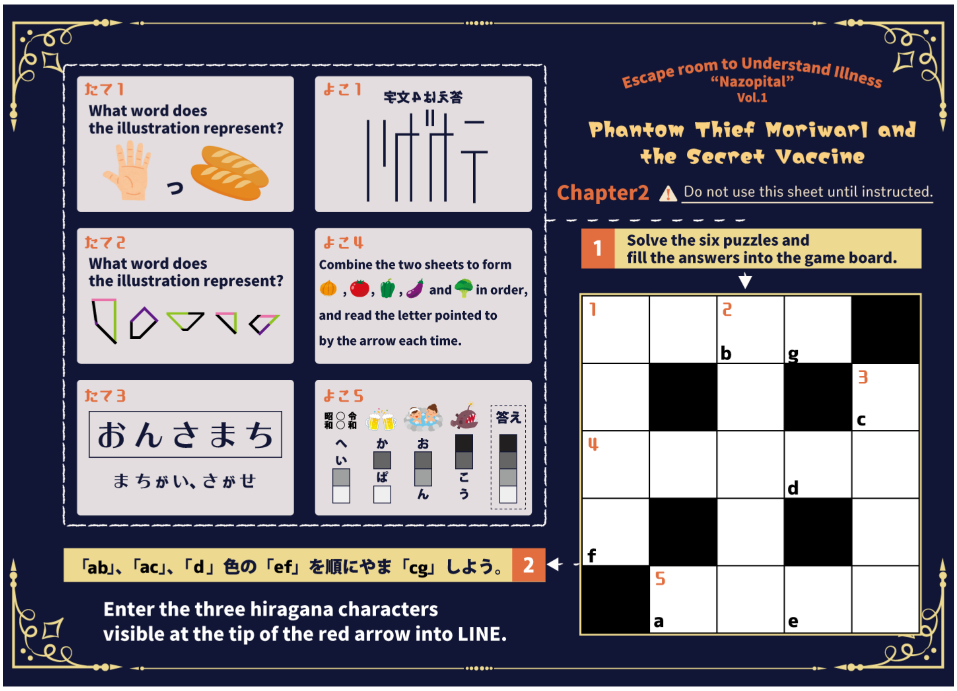

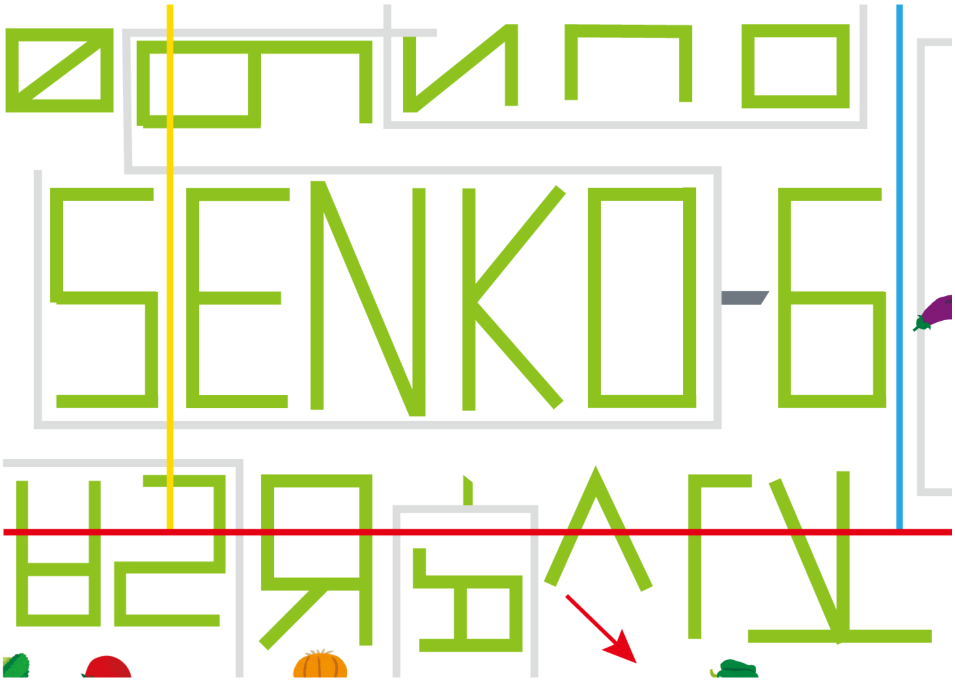

Table S1. The six cognitive process dimensions of Fink's taxonomy of significant learning [22].

Category of learning	Definition
Learning how to learn	Students will develop the ability to learn better, develop learning skills, in this course and in life in general.
Caring	Students will care more deeply about something, value or appreciate something.
Human dimension	Self: Students will better understand themselves, their identities, and their strengths and areas for growth.<br>Others: Students will be able to understand others, empathize with others, and interact positively and productively with others.
Integration	Students will be able to identify relationships, make connections between ideas within and outside of the course.
Application	Students will know how to "do" important tasks, apply knowledge, develop thinking abilities (critical, creative, practical thinking), or perform skills.
Foundational knowledge	Students will understand and remember key concepts, terms, relationships, facts, gain essential understandings.

Table S2. Item-level adjusted correct response rates immediately after the intervention (escape room vs lecture).

Question No.	Escape room EMM (95% CI)	Lecture EMM (95% CI)	Mean difference (95% CI)	p value
1	0.991 (0.974 to 1.007)	0.998 (0.979 to 1.017)	-0.007 (-0.032 to 0.018)	0.583
2	0.981 (0.958 to 1.003)	0.996 (0.970 to 1.021)	-0.015 (-0.049 to 0.019)	0.393
3	0.925 (0.879 to 0.971)	0.981 (0.928 to 1.033)	-0.056 (-0.126 to 0.015)	0.121
4	0.960 (0.923 to 0.997)	0.979 (0.936 to 1.021)	-0.018 (-0.075 to 0.039)	0.528
5	0.729 (0.658 to 0.800)	0.929 (0.848 to 1.010)	-0.200 (-0.308 to -0.092)	<0.001
6	0.928 (0.886 to 0.970)	0.991 (0.943 to 1.039)	-0.063 (-0.128 to 0.001)	0.053
7	0.767 (0.691 to 0.843)	0.938 (0.852 to 1.025)	-0.172 (-0.290 to -0.053)	0.005

Values are estimated marginal means (EMMs) from ANCOVA models adjusting for the corresponding pre-class item score. Mean difference is Escape Room minus Lecture.
